# PR55α regulatory subunit of PP2A inhibits the MOB1/LATS cascade and activates YAP in pancreatic cancer cells

**DOI:** 10.1038/s41389-019-0172-9

**Published:** 2019-10-28

**Authors:** Ashley L. Hein, Nichole D. Brandquist, Caroline Y. Ouellette, Parthasarathy Seshacharyulu, Charles A. Enke, Michel M. Ouellette, Surinder K. Batra, Ying Yan

**Affiliations:** 1 Department of Radiation Oncology, University of Nebraska Medical Center, Omaha, NE USA; 2 Department of Biochemistry and Molecular Biology, University of Nebraska Medical Center, Omaha, NE USA; 3 Department of Internal Medicine, University of Nebraska Medical Center, Omaha, NE USA

**Keywords:** Pancreatic cancer, Growth factor signalling

## Abstract

PP2A holoenzyme complexes are responsible for the majority of Ser/Thr phosphatase activities in human cells. Each PP2A consists of a catalytic subunit (C), a scaffold subunit (A), and a regulatory subunit (B). While the A and C subunits each exists only in two highly conserved isoforms, a large number of B subunits share no homology, which determines PP2A substrate specificity and cellular localization. It is anticipated that different PP2A holoenzymes play distinct roles in cellular signaling networks, whereas PP2A has only generally been defined as a putative tumor suppressor, which is mostly based on the loss-of-function studies using pharmacological or biological inhibitors for the highly conserved A or C subunit of PP2A. Recent studies of specific pathways indicate that some PP2A complexes also possess tumor-promoting functions. We have previously reported an essential role of PR55α, a PP2A regulatory subunit, in the support of oncogenic phenotypes, including in vivo tumorigenicity/metastasis of pancreatic cancer cells. In this report, we have elucidated a novel role of PR55α-regulated PP2A in the activation of YAP oncoprotein, whose function is required for anchorage-independent growth during oncogenesis of solid tumors. Our data show two lines of YAP regulation by PR55α: (1) PR55α inhibits the MOB1-triggered autoactivation of LATS1/2 kinases, the core member of the Hippo pathway that inhibits YAP by inducing its proteasomal degradation and cytoplasmic retention and (2) PR55α directly interacts with and regulates YAP itself. Accordingly, PR55α is essential for YAP-promoted gene transcriptions, as well as for anchorage-independent growth, in which YAP plays a key role. In summary, current findings demonstrate a novel YAP activation mechanism based on the PR55α-regulated PP2A phosphatase.

## Introduction

**Fig. 1:**
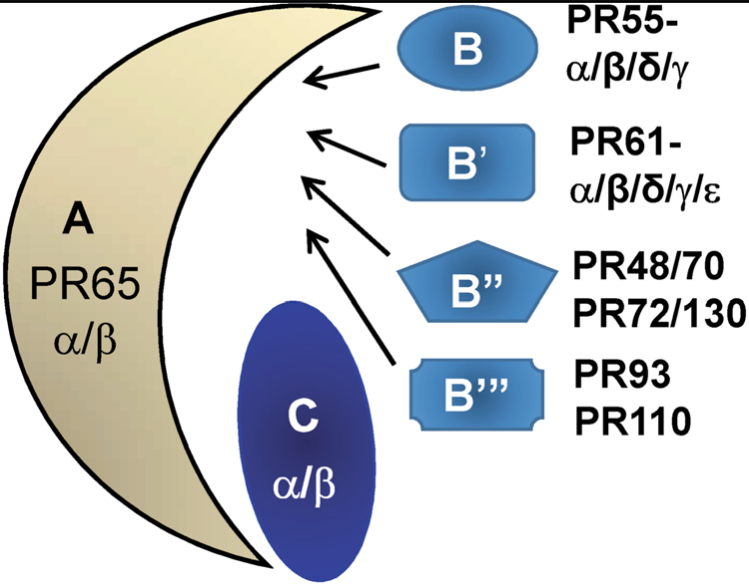
Protein phosphatase 2A (PP2A) heterotrimeric holoenzymes. One scaffold (PR65 or A) subunit binds to one catalytic (C) subunit to form an A/C heterodimer, which can further complex with one of the regulatory (B) subunits. The A and C subunits each contain two highly conserved isoforms (97% similarity between Cα and Cβ and 87% similarity between Aα and Aβ). A large number of the B subunits are classified into four distinct subfamilies (B, B′, B″, and B‴), which share no homology^1–3^

The PP2A (protein phosphatase 2A) family of hetero-trimers accounts for the majority of serine/threonine phosphatase activities in human cells^1,2^. Each PP2A consists of one catalytic subunit (C), one scaffolding subunit (A), and one regulatory subunit (B)^1,2^ (Fig. 1). While the A and C subunits each contain two highly conserved isoforms, a large number of the B subunits are classified into four distinct subfamilies (B, B′, B″, and B‴) and share no homology. It is the B subunit that determines the substrate specificities and cellular localizations of PP2A^1,3^.

PP2A regulates diversified cellular functions, including proliferation, migration, and survival, whereas its role in oncogenesis remains poorly defined^1,2,4–7^. Currently, PP2A is defined as a putative tumor suppressor, mostly based on the loss-of-function analyses using inhibitors for the highly conserved A/C subunits, such as okadaic acid (C-subunit inhibitor), short interfering RNA (siRNA) against the A/C subunits, or viral oncoproteins displacing the B subunits (e.g., SV40 small-t)^6,8,9^. In addition, PP2A-B subunits PR61 (α/γ) and PR72 act as tumor suppressors preventing oncogenic transformation^10–13^. In contrast, PP2A-B subunits PR130 and PR55α function as tumor-promoters^1,2,14^. While PR130 sustains EGF-mediated survival signaling and supports metastasis^15,16^, PR55α activates the Raf/MEK/ERK oncogenic cascade by dephosphorylating KSR-S392 and Raf-S259/S295, and stabilizes β-catenin and c-Myc oncoproteins by dephosphorylating β-catenin-T41/S37/S33 and c-Myc-T58, respectively^17–19^. Furthermore, we have reported the role of PR55α in the support of tumorigenicity and metastasis of pancreatic cancer cells^20^.

Yes-associated protein (YAP), a transcription coactivator, activates essential genes for tumorigenesis and metastasis of most solid tumors^21–24^. YAP is also required for KRAS-driven pancreatic tumorigenesis and compensates for KRAS-loss in the KRAS-addicted pancreatic cancer cells to produce malignant phenotypes^25–27^. YAP is inhibited by the Hippo tumor suppressor pathway that induces YAP phosphorylation at multiples sites, including YAP-S127 causing YAP cytoplasmic retention by 14-3-3, and YAP-S397 triggering YAP degradation by βTrCP-SCF^28^.

The Hippo pathway primarily consists of MST1/2, MOB1, and LATS1/2. Upon activation, MST1/2 phosphorylates LATS1-T1079/LATS2-T1041, while it concurrently autophosphorylates itself between the catalytic-domain and the SARAH-domain to create a MOB1-docking site for inducing MOB1-T12/T35 phosphorylation^29–31^, which subsequently triggers the autophosphorylation of LATS1-S909/LATS2-S872, leading to LATS1/2 autoactivation^31^.

The kinase roles in the Hippo pathway and YAP activation have been clearly elucidated, whereas the essential input of phosphatases in this regulation remains poorly understood. Previous studies implicate PP2A in the regulation of the Hippo pathway: while proteomics/RNAi screening show that dSTRIPAK-associated PP2A suppresses the Hippo pathway in Drosophila, PP2A inhibition by okadaic acid induces MOB1-phosphorylation in yeast cells and MST1/2-phosphorylation in HeLa cervical cancer cells^32–34^. The current study reveals a critical role of PR55α in the inhibition of the MOB1/LATS autoactivation loop and activation of YAP in pancreatic normal and cancer cells.

## Results

### PR55α supports the activation of YAP in pancreatic cancer cells

**Fig. 2:**
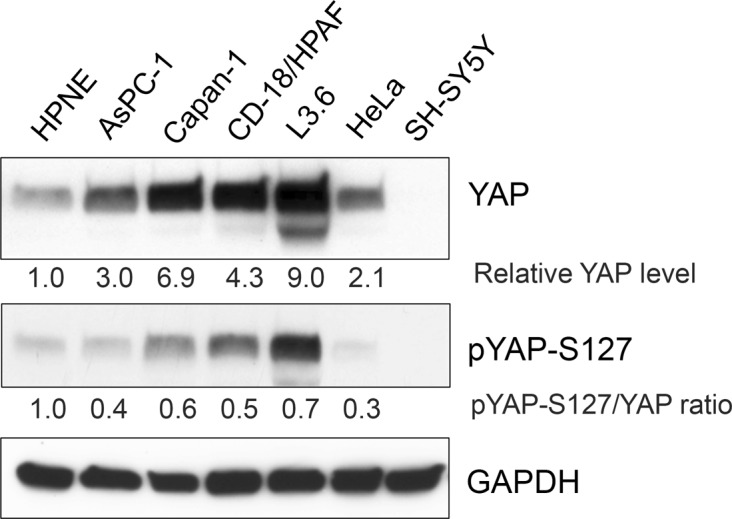
Analysis of YAP protein level and YAP-S127 phosphorylation in normal and malignant human pancreatic cells. YAP protein level is markedly increased in the human pancreatic cancer cells (AsPC-1, Capan-1, CD18/HPAF, and L3.6) compared with human pancreatic ductal cells (HPNE), as determined by immunoblotting. HeLa human cervical cancer cells and SH-SY5Y human neuroblastoma cells serve as a positive and negative control, respectively, for YAP protein expression. GAPDH in the lysates was measured as internal controls. The protein levels of YAP, pYAP-S127, and GAPDH were quantified using ImageJ software. Relative YAP and pYAP-S127 levels in the samples were normalized with GAPDH levels and the ratio of pYAP-S127/YAP determined

We have shown that PR55α supports anchorage-independent growth and tumorigenicity of pancreatic cancer cells^20^, which is also the best-known function of YAP in cancer^21,24^. Immunoblotting detected a marked increase in YAP level in human pancreatic cancer cells relative to the HPNE human normal pancreatic cells immortalized with telomerase^35^ (Fig. 2). In contrast, YAP-S127 phosphorylation, which induces YAP cytoplasmic retention by 14-3-3, is only moderately increased in pancreatic cancer cells compared with HPNE.

**Fig. 3:**
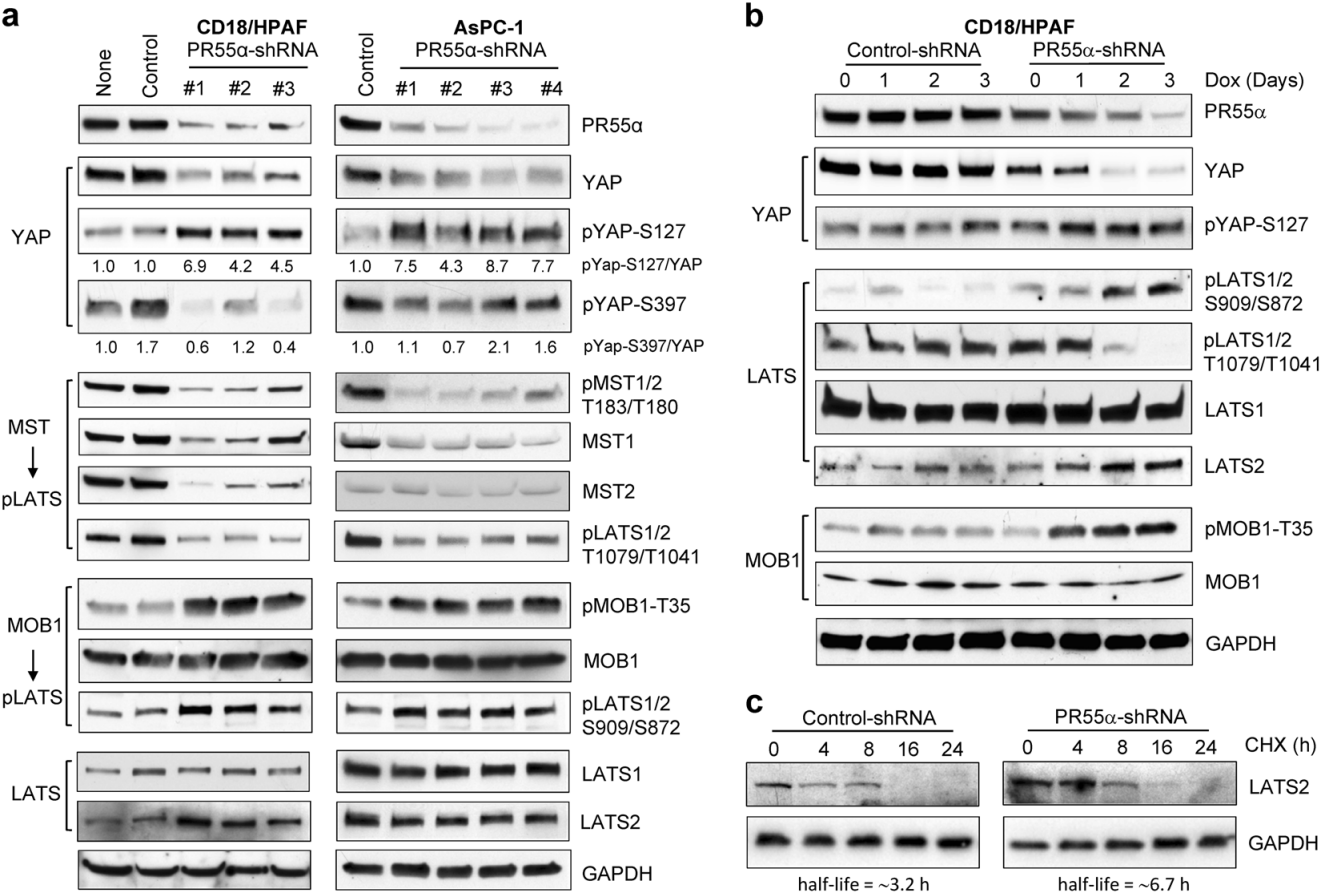
PR55α-knockdown by shRNA inhibits YAP and activates the MOB1-LATS1/2 cascade in pancreatic cancer cells. **a** CD18/HPAF and AsPC-1 cells were stably transduced with Dox-inducible shRNAs targeting various regions of PR55α or, as a control, with nontargeting shRNA. After incubation with Dox (2 µg/ml) for 3 days, the cell lysates (100 µg) were analyzed by immunoblotting for the indicated protein levels and/or phosphorylation. GAPDH served as an internal protein expression control. **b** Validation of the effects of PR55α on MOB1/LATS/YAP cascade in pancreatic cancer cells. shRNA-transduced CD18/HPAF cells were incubated with Dox (2 µg/ml) for the days indicated and analyzed for PR55α expression and the levels/phosphorylation of MOB1, LATS1/2, and YAP. GAPDH serves as an internal control. **c** To test the effect of PR55α on LATS2 protein stability, shRNA-transduced CD18/HPAF cells were incubated in medium containing cycloheximide (CHX, 15 μg/ml) to halt protein synthesis for the indicated hours. Whole-cell extracts were analyzed for LATS2 and GAPDH protein levels by immunoblotting. The protein levels of LATS2 and GAPDH were quantified using ImageJ software; relative LATS2 levels were normalized with GAPDH levels in the samples and protein half-life determined using SigmaPlot (version 11.2) analytical program

YAP inhibition by the LATS1/2 kinases is primarily via two mechanisms: phosphorylation of YAP-S397 leading to proteasomal degradation by β-TrCP and phosphorylation of YAP-S127 resulting in 14-3-3 cytoplasmic retention^36^. Therefore, we examined the impact of PR55α on YAP activation in pancreatic cancer cells using a series of Doxycycline (Dox)-inducible PR55α-shRNAs. As shown in Fig. 3a, PR55α-knockdown by short-hairpin RNAs (shRNAs) markedly decreased PR55α level in pancreatic cancer cells (CD18/HPAF and AsPC-1) compared with parental and Control-shRNA-transduced cells. Consequently, YAP level was largely reduced following PR55α-knockdown with a concomitant increase in YAP-S127 phosphorylation in the cells. On the other hand, the steady-state level of YAP-S397 phosphorylation was not particularly increased following PR55α-knockdown. Since YAP-S397 phosphorylation is specifically linked to YAP proteasomal degradation and cannot accumulate in the cells^37^, this outcome was anticipated. Collectively, these results suggest a role of PR55α in the maintenance of YAP protein and dephosphorylation of YAP-S127 in pancreatic cancer cells.

### The effects of PR55α on the Hippo tumor suppressor pathway in pancreatic cancer cells

The Hippo pathway negatively regulates YAP level and activity. Therefore, we examined the effects of PR55α on the core members of this pathway (MST1/2, MOB1, and LATS1/2) in pancreatic cancer cells using Dox-inducible shRNAs.

We tested the effect of PR55α on the MST/LATS cascade in CD18/HPAF and AsPC-1 cells. Unexpectedly, PR55α-knockdown by shRNA resulted in decreases in MST1/2 levels and phosphorylation, along with the phosphorylation of its downstream targets LATS1-T1079/LATS2-T1041 (Fig. 3a).

Next, we analyzed the role of PR55α in the MOB1-triggered autophosphorylation of LATS1-S909/LATS2-S872, the key step leading to the LATS1/2 autoactivation loop^37^. In both CD18/HPAF and AsPC-1 cells, knockdown of PR55α induced the phosphorylation of MOB1-T35 and LATS1-S909/LATS2-S872 (MOB1 downstream targets), which indicates activation of the MOB1/LATS autoactivation loop^28,38^ (Fig. 3a). While LATS1 protein level remained unchanged, LATS2 protein was increased following PR55α-knockdown in CD18/HPAF cells. However, this effect was not detected in AsPC-1 cells. This difference probably attributes to cell-type specificity, as CD18/HPAF cells originated from metastatic liver lesions, while AsPC-1 cells were isolated from ascites^39^.

We validated the impact of PR55α on the MOB/LATS/YAP cascade with a time-course study. Following PR55α-knockdown by shRNA in CD18/HPAF cells, there was a time-dependent decrease in YAP level with a concurrent increase in YAP-S127 phosphorylation (Fig. 3b). Consistently, these changes in YAP were tightly associated with an increase in phosphorylation of both LATS1-S909/LATS2-S872 and MOB1-T35, an increase of protein level of LATS2, and the diminution of LATS1-T1079/LATS2-T1041 phosphorylation (Fig. 3b).

Since LATS2 was reported to be regulated by proteasomal degradation^40^, we evaluated the effect of PR55α on LATS2 protein stability using protein synthesis inhibitor cycloheximide (CHX), as described in our study^41^. The analysis indicated that LATS2 protein half-life is ~3.2 h in CD18/HPAF cells, while it elongated to ∼6.7 h after the knockdown of PR55α (Fig. 3c).

Collectively, these results suggest an essential role for PR55α in the inhibition of the MOB1/LATS cascade that directly prevents YAP activation.

### Ectopic PR55α expression induces YAP activation in normal human pancreatic ductal cells

**Fig. 4:**
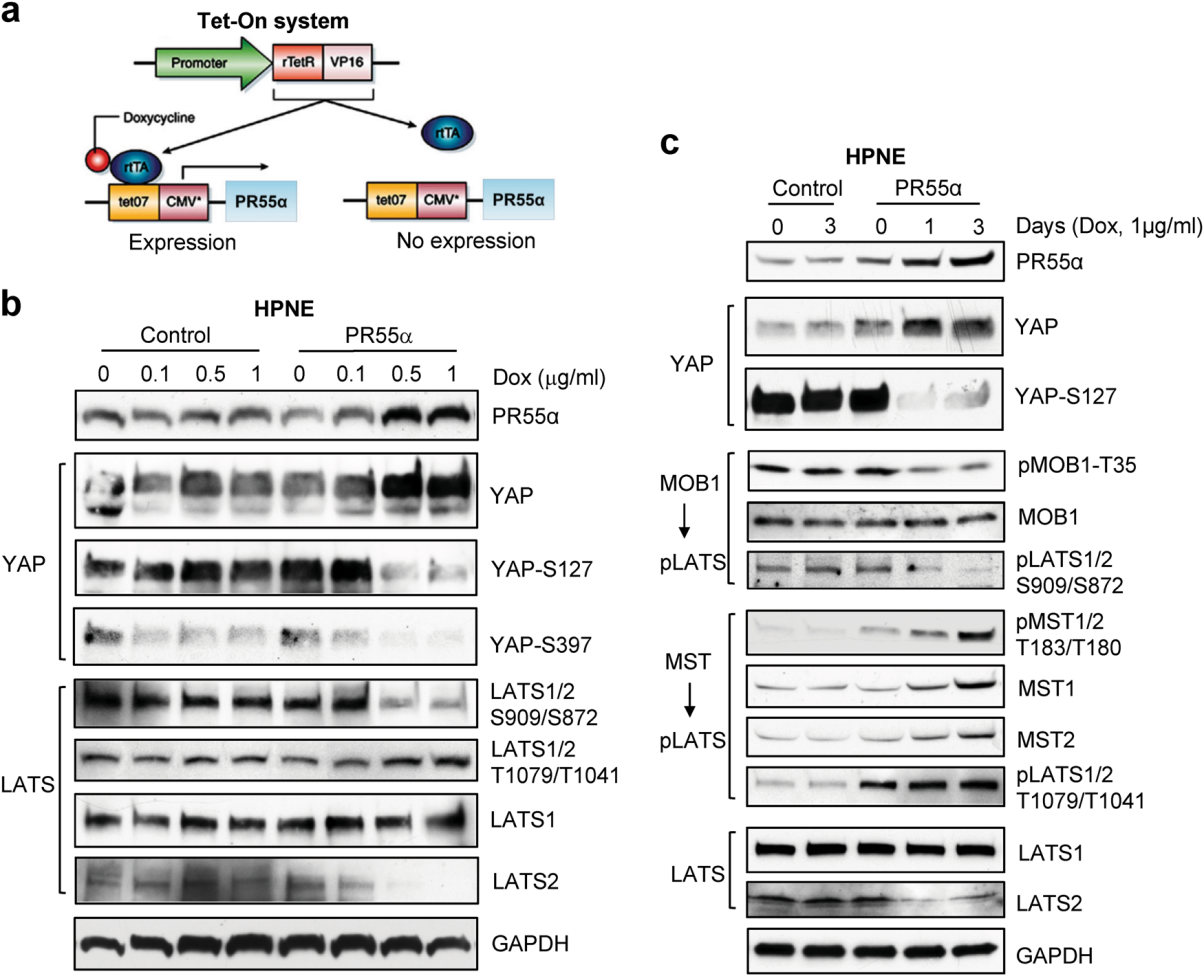
Ectopic PR55α expression in human normal pancreatic ductal cells (HPNE) activates YAP and inhibits the MOB1-LATS1/2 cascade. **a** Dox-inducible retroviral vector expressing PR55α (pRevTRE-PR55α) was constructed and retrovirus was produced as described in the “Materials and methods“ section. Subsequently, HPNE cells were transduced with both pRevTet-On (Clontech) that expresses rtTA and pRevTRE-PR55α (or pRevTRE-Control) and selected for resistant cells to both G418 (400 µg/ml) and Hygromycin B (200 µg/ml). Diagram demonstrates the Tet-inducible retroviral vector (pRevTRE-PR55α) expressing PR55α. **b** The transduced cells were induced for ectopic PR55α expression by incubation with increasing doses of Dox (1 µg/ml) for 3 days and the effect of PR55α on the phosphorylation and level of YAP and LATS1/2 analyzed by immunoblotting. GAPDH served as an internal control. **c** Control- and PR55α-transduced HPNE cells were incubated with Dox (1 µg/ml) for the indicated days and analyzed for the effect of PR55α on YAP and the Hippo pathway (MST1/2, MOB1, and LATS1/2), by analyzing their phosphorylation and levels by immunoblotting. GAPDH served as an internal control

To define the role of PR55α in normal pancreatic cells, we constructed the pREV-TRE-PR55α retroviral vector expressing Dox-inducible PR55α, which was further stably introduced into HPNE normal pancreatic ductal cells (Fig. 4a). Following 48 h induction with increasing doses of Dox, a marked increase in PR55α protein was detected in the pREV-TRE-PR55α-transduced cells but not in the control-vector-transduced cells (Fig. 4b). Associated with the PR55α induction were a concomitant increase of YAP protein levels and a simultaneous diminution of YAP-S127/S397 phosphorylation (Fig. 4b). In contrast, control-vector-transduced cells receiving the same treatment showed little effect on either PR55α expression or on YAP protein level/phosphorylation.

### Effects of ectopic PR55α on MOB1/LATS cascade in normal pancreatic ductal cells

We next examined the effect of PR55α on LATS1/2 phosphorylation and levels in HPNE cells. Ectopic PR55α expression in HPNE cells resulted in a marked decrease in LATS1-S909/LATS2-S872 phosphorylation along with a moderate increase in LATS1-T1079/LATS2-T1041 phosphorylation (Fig. 4b). Furthermore, LATS2 protein level was reduced in HPNE cells upon PR55α overexpression, while the effect is absent in the control cells (Fig. 4b). With a time-course study, we verified this effect of PR55α. Figure 4c shows that, following PR55α induction by Dox in HPNE cells, YAP protein level was increased, along with concomitant reductions in phosphorylation of YAP-S127, MOB1-T35, and LATS1-S909/LATS2-S872, and a decrease in LATS2 protein level. Immunoblotting also detected an increase in MST1/2 phosphorylation and level and LATS1-T1079/LATS2-T1041 phosphorylation (MST1/2 substrates) in HPNE cells.

Collectively, the results from both normal and malignant cells suggest a role of PR55α in YAP activation that involves the suppression of the MOB1/LATS autoactivation loop, leading to YAP phosphorylation/inhibition. Furthermore, this role of PR55α apparently does not require the MST/LATS cascade, since its activity increases in response to PR55α overexpression. This may implicate a feedback loop activation by PR55α.

### PR55α supports YAP protein stability

**Fig. 5:**
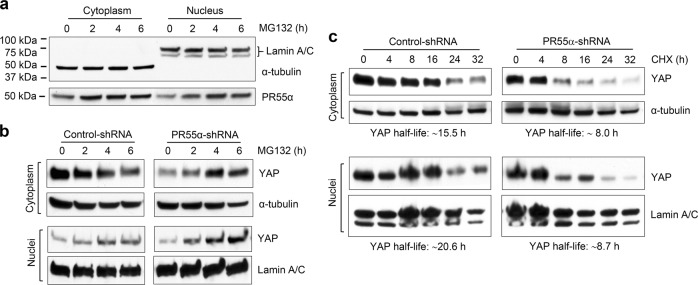
PR55α is essential for maintaining YAP stability. Cytoplasmic and nuclear extracts were isolated using the NE-PER™ Nuclear and Cytoplasmic Extraction Reagents (Fisher Scientific) and analyzed for YAP protein expression. **a** α-tubulin and Lamin A/C were used as the cytoplasmic and nuclear markers, respectively. PR55α in cytoplasm and nucleus was analyzed by Western blot analysis. **b** To inhibit cellular proteasome activity, CD18/HPAF cells transduced with Control-shRNA or PR55α-shRNA were treated with MG132 (25 μM) for the indicated hours. Cytoplasmic and nuclear extracts were isolated from the treated cells and analyzed for YAP expression by immunoblotting. α-tubulin and Lamin A/C served as the internal controls for cytoplasmic and nuclear extract, respectively. **c** To test the effect of PR55α on YAP protein stability, Control-/PR55α-shRNA-transduced CD18/HPAF cells were treated with CHX (15 μg/ml) to halt protein synthesis for the indicated hours, cytoplasmic and nuclear extracts were isolated from the treated cells, and YAP protein levels analyzed by immunoblotting. α-tubulin and Lamin A/C served as internal controls for cytoplasmic and nuclear extract, respectively

One of the primary mechanisms by which the Hippo pathway inhibits YAP is to induce its proteasomal degradation^42^. We, therefore, assessed the effect of PR55α on YAP protein stability in both the cytoplasm and nuclei of CD18/HPAF cells using α-tubulin and Lamin A/C as cytoplasmic and nuclear markers, respectively^43^. Immunoblotting showed that PR55α is ubiquitously expressed in both cytoplasm and nuclei of the cells and its level increases following proteasomal inhibition by MG132 (Fig. 5a). To test whether the decrease of YAP in PR55α-knockdown cells was due to proteasomal degradation, MG132 was used to block proteasome activity and assessed for its effect on YAP level. As shown in Fig. 5b, MG132 treatment of the PR55α-knockdown cells resulted in an induction of both cytoplasmic and nuclear YAP, while MG132 treatment of Control-shRNA knockdown cells caused a decrease in cytoplasmic YAP level and only a subtle increase in the nuclear YAP. This suggests that PR55α inhibits YAP proteasomal degradation in CD18/HPAF cells.

We tested the effect of PR55α on YAP protein half-life in the cytoplasm and nuclei of CD18/HPAF cells, which were treated with CHX to block protein synthesis, and analyzed for YAP protein decay over time by western blot. The results show that YAP protein half-life was ~15.5 and 20.6 h in the cytoplasm and nuclei of the control cells, respectively, whereas it was only ∼8 and ∼8.7 h in the cytoplasm and nuclei of the PR55α-knockdown cells, respectively (Fig. 5c). These findings suggest that PR55α supports YAP protein stability via a mechanism that involves the inhibition of the proteasomal degradation of YAP.

### Interaction of PR55α and YAP in pancreatic normal and cancer cells

**Fig. 6:**
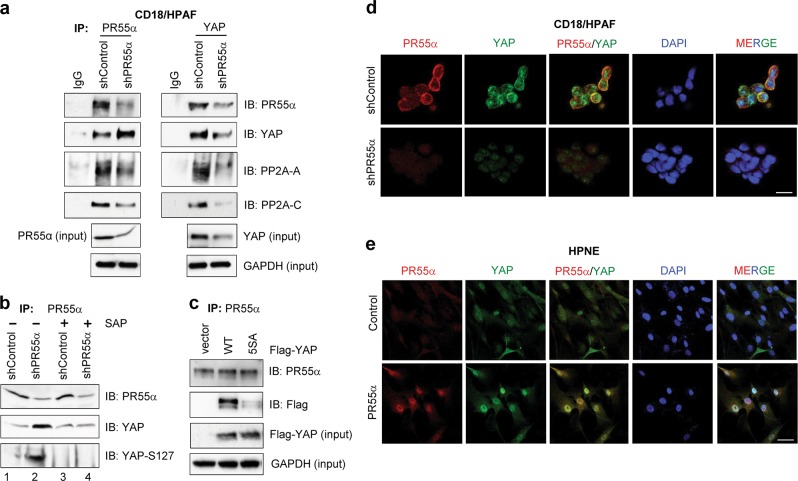
Interaction of PR55α and YAP in pancreatic malignant and normal cells. **a** PR55α was immunoprecipitated from 1 mg protein lysates of CD18/HPAF cells expressing Control-shRNA or PR55α-shRNA with anti-PR55α (100C1) rabbit IgG and probed by immunoblotting for the presence of PR55α, PP2A-C, and PP2A-A subunits with anti-PR55α (2G9), anti-PP2A-C (ID6), and anti-PP2A-A (H300) antibody, respectively. YAP and GAPDH in the lysate were measured by immunoblotting for YAP protein loading control and internal control for protein quantification, respectively. **b** PR55α was immunoprecipitated from the indicated protein lysates (1 mg per sample) with anti-PR55 (100C1) antibody. The obtained immunoprecipitates were divided into two halves: one half remained untreated (−) and the other half was treated with Shrimp Alkaline Phosphatase (SAP) at 10 units/ml (+) at 37 °C for 1 h. The resulting precipitates were rinsed once with cell lysis buffer and subjected to immunoblotting analysis for PR55α, YAP, and YAP-S127 with the antibody for PR55α (2G9), YAP (D24E4), and YAP-Ser127 phosphorylation (D9W2I), respectively. **c** PR55α were immunoprecipitated (IP) with anti-PR55α (2G9) antibody from CD18/HPAF cells stably transduced with empty vector, Flag-YAP, or Flag-YAP (5SA) mutant and immunoblotted (IB) using anti-PR55α (100C1) and anti-Flag (M2) antibodies. Lysates from the indicated cells were probed for Flag-YAP (input) and GAPDH (input) with specific antibodies by Western blotting. Intracellular distribution and co-localization of PR55α and YAP in pancreatic malignant and normal cells. The indicated cells were induced for the expression of PR55α-shRNA (CD18/HPAF) (**d**) or ectopic PR55α (HPNE) (**e**) by 2 µg/ml Dox for 3 days, and stained with anti-PR55α (100C1) and anti-YAP (1A12) antibodies, as described in the “Materials and methods” section. Images were analyzed for the cellular distribution of PR55α and YAP using a Zeiss-810 confocal laser-scanning microscope. Co-localization of PR55α and YAP in CD18/HPAF cells with/without PR55α-knockdown and in HPNE cells with/without ectopic PR55α expression were examined and shown as merged images (*PR55α/YAP* and *MERGE*). Scale bars, 50 µm

By reciprocal co-immunoprecipitation assay described previously^41^, we examined the interaction of PR55α and YAP in CD18/HPAF cells. As a control, immunoprecipitation with nonimmunized IgG was included in the study. Immunoblotting revealed the presence of both PR55α and YAP, along with PP2A-A and PP2A-C subunits in the immunoprecipitates obtained either with anti-PR55α or anti-YAP antibody (Fig. 6a). Furthermore, relative to control cells, PR55α-knockdown cells displayed a lesser amount of PR55α, PP2A-C, and PP2A-A subunits in the immunoprecipitates obtained with anti-PR55α or anti-YAP antibody (Fig. 6a). Unexpectedly, relative to control cells, a higher level of YAP was detected in the anti-PR55α immunoprecipitates obtained from PR55α-knockdown cells, which is inconsistent with a lesser amount of YAP revealed in the anti-YAP immunoprecipitates obtained from PR55α-knockdown cells relative to control cells (Fig. 6a).

To determine whether the high YAP level present in the anti-PR55α immunoprecipitates from PR55α-knockdown cells could be attributed to YAP hyperphosphorylation, the anti-PR55α immunoprecipitates from control and PR55α-knockdown cells were treated with/without shrimp alkaline phosphatase (SAP) and analyzed for PR55α and p-YAP/YAP levels. As shown in Fig. 6b, the high YAP level in the anti-PR55α immunoprecipitates from PR55α-knockdown cells was markedly diminished after SAP treatment (*YAP*, lane 4 vs. 2). Immunoblotting of YAP-Ser127 phosphorylation confirmed the effectiveness of SAP in YAP dephosphorylation (*YAP-S127*, lane 4 vs. 2).

We compared the interaction of PR55α with YAP [wild-type (WT)] versus YAP(5SA) (constitutive active mutant), in which the LATS phosphorylation sites (S61/S109/S127/S164/S397) were mutated to alanine^38,42^. CD18/HPAF cells were stably transduced with Flag-YAP(WT) or Flag-YAP(5SA) and immumonoprecitated with anti-PR55α antibody. As shown in Fig. 6c, anti-Flag antibody detected the presence of Flag-YAP and Flag-YAP(5SA) in the anti-PR55α immunoprecipitates obtained from their respective lysate, whereas the Flag-YAP(WT) level was 28-fold higher than Flag-YAP(5SA). This result implicates a direct interaction of PR55α and YAP and further supports that PR55α expresses a higher affinity toward phosphorylated YAP.

Using immunofluorescence (IF) confocal microscopy, we analyzed the intracellular level, distribution, and colocalization of PR55α and YAP. The results show the detection of PR55α and YAP in both the cytoplasm and nucleus of CD18/HPAF cells, with PR55α slightly more concentrated in the cytoplasm and YAP slightly more in the nucleus (*shControl*, Fig. 6d and Supplementary Fig. S1a). In the PR55α-knockdown cells, both PR55α and YAP levels were markedly reduced and the residual YAP was now mainly present in the nucleus (*shPR55α*, Fig. 6d, and Supplementary Fig. S1a).

We next analyzed the intracellular distribution of PR55α and YAP in normal HPNE cells. Both PR55α and YAP were also detected in both the cytoplasm and nucleus of the cells (Fig. 6e and Supplementary Fig. S1b). Upon ectopic PR55α expression, YAP and PR55α protein levels were concurrently increased in the cells and the additional amounts of the proteins were predominantly detected in the nuclei.

Co-localization studies revealed that, in both CD18/HPAF and HPNE cells, there was a significantly greater co-localization of PR55α and YAP in the PR55α-high cells (CD18/HPAF-shControl and HPNE-PR55α) compared with their respective isogeneic PR55α-low cells (CD18/HPAF-shPR55α and HPNE-PR55α) (Fig. 6d–e and Supplementary Fig. S1c).

Collectively, these results suggest a physical interaction and functional relationship of PR55α and YAP in pancreatic cancer and normal cells.

### Effect of MOB1 on the PR55α-promoted YAP activation

MOB1-triggered LATS1-S909/LATS2-S872 autophosphorylation is the key event resulting in YAP inhibition^38,44^. The results in Figs. 3–4 demonstrate that PR55α-promoted YAP activation in both malignant and normal cells is inversely associated with the activity of the MOB1/LATS axis. We, therefore, probed the role of MOB1 in YAP activation by PR55α using siRNA. MOB1 exists in two isoforms, MOB1A and MOB1B, which share 95% protein sequence identity and are functionally redundant^44^. Since there is no antibody available to distinguish MOB1A and MOB1B, we analyzed their expressions by RT-PCR in a panel of pancreatic normal and cancer cells, which showed that MOB1A mRNA level is 5–15 fold higher than MOB1B in these cells (Supplementary Fig 2a, b). This result was confirmed by siRNA-knockdown studies, which showed that MOB1A-siRNA but not MOB1B-siRNA effectively reduced the total MOB1 protein level in the cells (Supplementary Fig 2c).

**Fig. 7:**
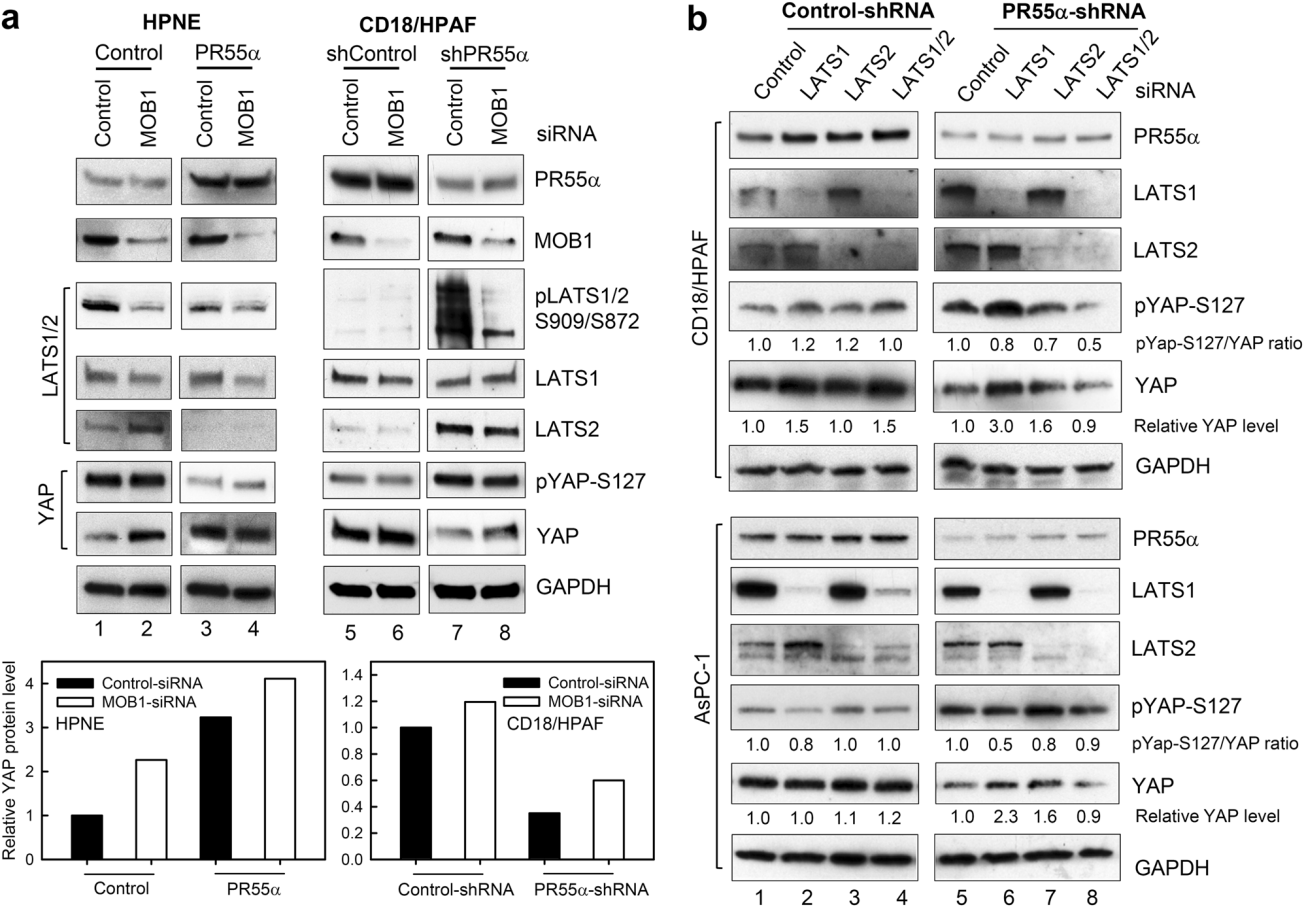
Effects of MOB1- and LATS1/2-knockdown by siRNA on the YAP activation promoted by PR55α. **a** MOB1 was knocked down by siRNAs in both HPNE and CD18/HPAF cells with/without the expression of ectopic PR55α and PR55α-shRNA, respectively. After 72 h siRNA-transfection, the cells were analyzed for the phosphorylation and/or level of MOB1, LATS1/2, and YAP by immunoblotting. GAPDH served as an internal control. The levels of YAP and GAPDH were quantified using ImageJ software. YAP protein levels were normalized by the corresponding GAPDH levels and relative YAP levels in the samples were analyzed by SigmaPlot software and presented as bar graphs. **b** LATS1 and/or LATS2 were knocked down by siRNAs in CD18/HPAF and AsPC-1 cells expressing Control-shRNA or PR55α-shRNA. After 72 h incubation in medium containing Dox (2 µg/ml) to maintain shRNA expression, the cells were analyzed for the phosphorylation and/or levels of PR55α, LATS1, LATS2, YAP, and GAPDH. The levels of YAP and GAPDH were quantified using ImageJ software and relative YAP protein levels versus GAPDH levels were indicated under the YAP blots

With MOB1A-siRNA, we assessed the role of MOB1 in the regulation of LATS1/2 and YAP by PR55α. MOB1-knockdown in HPNE-Control cells resulted in a decrease of LATS1-S909/LATS2-S872 phosphorylation and a concurrent increase in YAP protein level, as shown in Fig. 7a (lane 2 vs. 1, Bar graph). Similarly, MOB1-knockdown in HPNE-PR55α cells caused a marginal decrease in the already low LATS1-S909/LAT2-S872 phosphorylation and increase of YAP level compared with the HPNE-PR55α cells with control-knockdown (Fig. 7a, lane 4 vs. 3, Bar graph). However, MOB1-knockdown in HPNE cells produced no effect on YAP-S127 phosphorylation or LATS2 protein levels, which are negatively affected only by PR55α level.

We next evaluated the effect of MOB1 on the regulation of LATS and YAP by PR55α in malignant CD18/HPAF cells. While MOB1-knockdown by siRNA had very little effect on the low level of LATS1-S909/LATS2-S872 phosphorylation in CD18/HPAF cells, it markedly diminished the induction of LATS1-S909/LATS2-S872 phosphorylation caused by the PR55α-knockdown in CD18/HPAF cells (Fig. 7a, right panel, bar graph). In both control- and PR55α-shRNA-transduced CD18/HPAF cells, MOB1-knockdown by siRNA resulted in a subtle increase of YAP protein level but had little effect on YAP-S127 phosphorylation, or LATS2 protein level (Fig. 7a, lane 6 vs. 5 and lane 8 vs. 7), all of which were significantly affected by the level of PR55α, (Fig. 7a, right panel, lanes 7–8 vs. lanes 5–6).

These results indicate that PR55α suppresses the activation of MOB1/LATS cascade, while MOB1-inhibition cannot fully compensate for the loss of PR55α to restore YAP activation, suggesting that PR55α holds a dominant control on the magnitude of YAP activation.

### Effect of LATS1/2 in the PR55α-promoted YAP activation

We investigated the role of LATS1/2 in the activation of YAP by PR55α in CD18/HPAF and AsPC-1 pancreatic cancer cells. In control-shRNA-transduced cells, knockdown of LATS1 and/or LATS2 by siRNA had only subtle effects on YAP phosphorylation and level in the cells (Fig. 7b, *Control-shRNA*). In PR55α-shRNA-transduced cells, knockdown of LATS1 or LATS2 alone by siRNA resulted in 1.6-3 fold increases in YAP protein levels relative to control cells (Fig. 7b, *YAP*, lanes 6–7 vs. lane 5). However, inhibition of both LATS1 and LATS2 by siRNA in the PR55α-knockdown cells resulted in a subtle, if any, decrease in YAP level compared with control cells (Fig. 7b, *YAP*, lane 8 vs. 5). Thus, in the PR55α-high (*Control-shRNA*) cells, manipulation of LATS1/2 levels apparently produced little effect on YAP level, whereas knockdown of either LATS1 or LATS2 in the PR55α-low (PR55α-shRNA) cells resulted in increases in YAP levels (Fig. 7b).

We also analyzed the effect of LATS1/2 on YAP-S127 phosphorylation in the presence/absence of PR55α-knockdown in pancreatic cancer cells. As shown in Fig. 7b, knockdown of LATS1 and/or LATS2 by siRNA had little effect on YAP-S127 phosphorylation in Control-shRNA-transduced cells, while it resulted in decreases in YAP-S127 phosphorylation in PR55α-shRNA-transduced cells. Furthermore, while knockdown of both LATS1 and LATS2 displayed an additive effect on inhibition of YAP-S127 phosphorylation in CD18/HPAF cells, this effect was not observed in AsPC-1 cells. These results suggest that PR55α plays a domainant role in the negative regulation of YAP-S127 phosphorylation.

### PR55α enhances YAP-targeted gene transcriptions and anchorage-independent growth

**Fig. 8:**
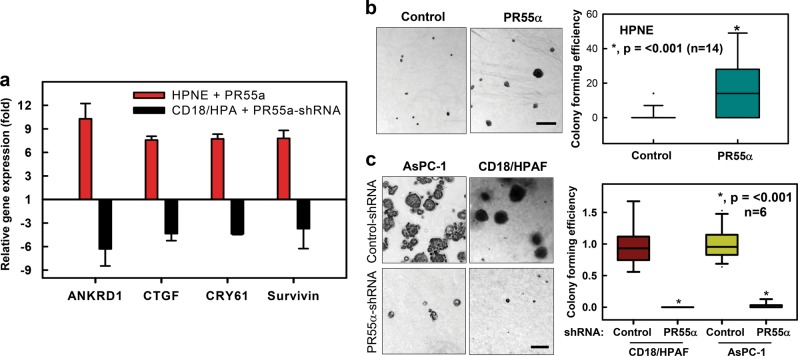
Effects of PR55α on YAP-targeted gene expressions and anchorage-independent growth. **a** PR55α promotes gene expressions of YAP targets. Ectopic PR55α and PR55α-shRNA were induced by Dox in HPNE for 3 days and CD18/HPAF cells for 6 days, respectively. The resulting cells were harvested to extract total RNA samples to analyze the mRNA expression of YAP target genes (ANKD1, CTGF, CRY61, and Survivin) by qRT-PCR, as described in the “Materials and methods” section. The study was repeated two times with duplicate samples and the result expressed as mean ± s.d (*n* = 6), *p* = 0.02. Statistical analyses were performed using the Student’s *t*-test. qRT-PCR, quantitative (q) Reverse Transcription (RT) PCR. **b** PR55α promotes anchorage-independent growth of HPNE cells. Upper panel: HPNE cells (5 × 10^4^) with/without ectopic PR55α expression were incubated with 1 µg/ml Dox for 48 h, plated in soft-agar in six-well plates and incubated for 14 days. Left panels: representative images of the soft-agar assay photographed by phase-contrast optics. Box plot: colonies in soft-agar were counted by ImageJ software and shown as mean ± s.d. of 14 samples. Scale bar represents 100 μm. **c** PR55α-knockdown by shRNA inhibits anchorage-independent growth of pancreatic cancer cells. shRNA-transduced CD18/HPAF and AsPC-1 cells were incubated with 2 µg/ml Dox for 48 h, plated in soft-agar in six-well plates at 4 × 10^4^ and incubated for 14 days. Left panels: representative images of soft-agar assay photographed by phase-contrast optics. Box plot: colonies in soft-agar were quantified by ImageJ software and shown as mean ± s.d. of two sets of experiments in triplicate samples. Scale bar represents 100 μm

To evaluate the biological significance of PR55α in promoting YAP activation, we analyzed the effect of PR55α on YAP-targeted gene expressions in HPNE (normal) and CD18/HPAF (malignant) cells. Real-time (RT)-PCR analyses revealed that PR55α expression was positively associated with YAP-activated transcriptions of *ANKRD1*, *CTGF*, *CYR61*, and *Survivin*^37^ in both HPNE and CD18/HPAF cells (Fig. 8a). Thus, ectopic PR55α expression in HPNE cells resulted in 8–10 fold increases in mRNA expressions of the YAP targets compared with control cells (red bars), while PR55α-knockdown by shRNA in CD18/HPAF cells caused a 4–6-fold reduction in mRNA levels of the YAP targets relative to the control-shRNA-transduced cells (black bars). These functional data confirm the role of PR55α in the promotion of YAP activation.

Promoting anchorage independence is the predominant role of YAP in oncogenesis^45–47^. YAP alone has been shown to induce anchorage-independent growth of HPNE normal cells by the soft-agar assay^22^. Therefore, we tested the effect of PR55α on anchorage-independent growth using the soft-agar assay^48^. The results in Fig. 8b show that, following Dox-induced ectopic PR55α expression, there was a significant induction of the proliferation of HPNE normal cells in soft-agar, indicative of anchorage-independent growth. Conversely, PR55α-knockdown by shRNA abrogated the clonogenicity of pancreatic cancer cells in soft-agar, indicative of loss of anchorage independence (Fig. 8c). These results support a critical role of PR55α in the positive regulation of YAP oncogenic function.

**Fig. 9:**
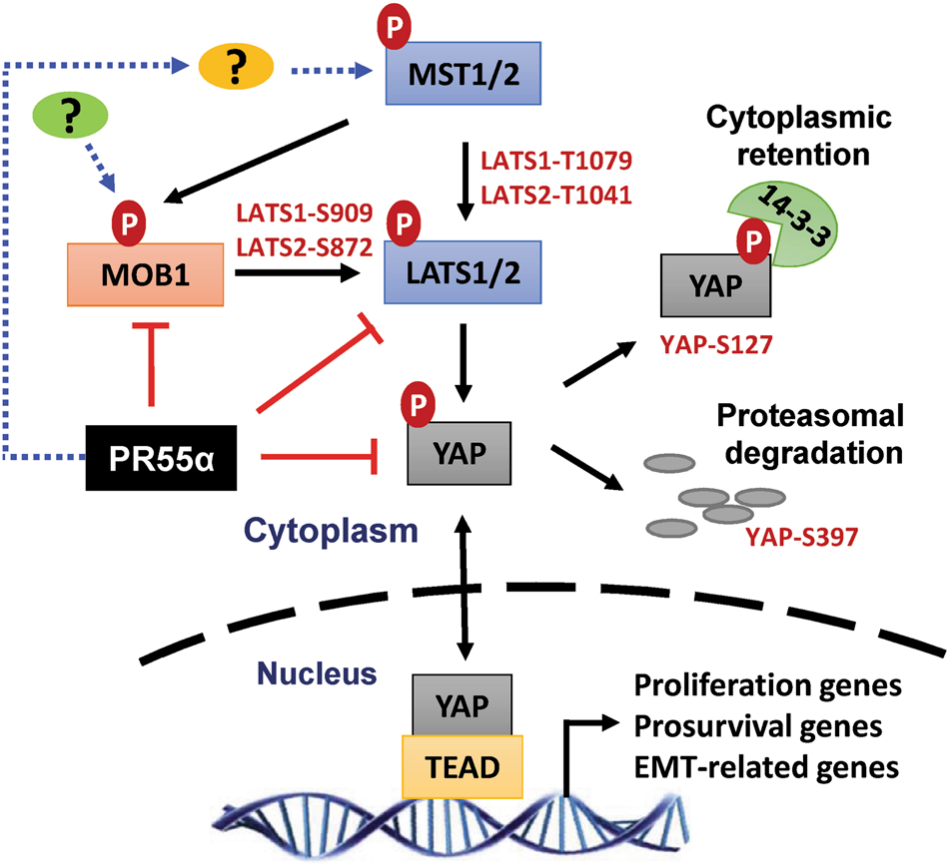
A model for the regulation of the Hippo pathway and YAP by PR55α. Black lines indicate a current understanding of the Hippo signaling cascades that regulate YAP phosphorylation and stability, resulting in YAP cytoplasmic retention by 14-3-3 and proteasomal degradation by SCF(β-TrCP). Red lines indicate the novel findings presented in this report, showing that PR55α inhibits the MOB1-activated LATS1-S909/LATS2-S872 autophosphorylation that prevents YAP activation, while PR55α concomitantly inhibits YAP phosphorylation, both of which lead to YAP activation. Blue dotted lines indicate that PR55α activates MST1/2 levels and phosphorylation through an unknown mechanism

In summary, the results of the current study (Figs. 2–8) reveal a novel mechanistic role of PR55α regulated PP2A in the activation of YAP oncoprotein. Figure 9 outlines the findings of this investigation, which indicates that PR55α specifically suppresses the MOB1-mediated LATS autophosphorylation/activation, which would otherwise promote YAP proteasomal degradation by β-TrCP and cytoplasmic retention by 14-3-3^37^. Furthermore, PR55α also exhibited a Hippo pathway-independent role in YAP activation, as siRNA-knockdown of either MOB1 or LATS1/2 did not compensate completely for the effect of PR55α-loss or PR55α-overexpression on YAP activation in both normal and malignant pancreatic cells (see Fig. 7), which suggests a regulation of YAP activation directly by PR55α or by another unknown mechanism regulated by PR55α.

## Discussion

PP2A has been suggested in the regulation of the Hippo pathway and YAP activation^32–34^, while the specific PP2A holoenzyme(s) involved were not identified. We recently identified the PR55α regulatory subunit of PP2A in the support of anchorage independence and tumorigenicity of pancreatic cancer cells, which coincidentally is the main function of YAP^20,49^. Thus, we investigated the role of PR55α in the regulation of the Hippo pathway and YAP activation in pancreatic cancer cells.

The results in this report elucidate a novel YAP activation mechanism based on the PR55α-regulated PP2A (Fig. 9), which engages PR55α at three levels of regulation^1^: inhibition of MOB1-triggered LATS1/2 autoactivation loop (LATS1-S909/LATS2-S872)^2^, destabilizing LATS2 protein and^3^ direct YAP activation (see Figs. 3–7). However, this PR55α-dependent mechanism of YAP activation apparently also activates MST1/2 (see Figs. 3–4), which may be through a feedback mechanism. Thus, the increase in MOB1-T35 phosphorylation in the PR55α-knockdown cells may attribute to the inhibition of the PR55α/PP2A phosphatase activity rather than the increase of MST1/2 kinase activity. Furthermore, MST1/2 level/activity is negatively associated with LATS2 protein stability in both HPNE normal and CD18/HPAF malignant cells in response to PR55α manipulation, implicating cross-talking or a feedback regulation mechanism among the Hippo pathway components.

PR55α-knockdown results in an increase of LATS2 stability in CD18/HPAF but not in AsPC-1 pancreatic cancer cells (see Fig. 3). Although the exact mechanism causing this difference is unclear, it is likely to be cell-type specific, as the two pancreatic cancer cell lines were originated from different metastatic sites, CD18/HPAF from the liver and AsPC-1 from ascites^39^. In order to metastasize and recolonize at distant organ sites, primary cancer cells need to adapt and survive a cascade of the environmental challenges by undergoing the processes of invasion→ intravasation→ systemic transport→ extravasation→ distant colonization^50^. Thus, pancreatic cancer cells metastasizing to the liver versus ascites would have gone through very different adaptive processes and LATS2 stability regulation could be one of those mechanisms needing to be altered to fit different processes. Future studies will be needed to elucidate the mechanism and biological significance of LATS2 regulation during metastasis.

Although PR55α inhibits the MOB1-triggered LATS1/2 autoactivation that blocks YAP, knockdown of MOB1 in the PR55α-high cells (HPNE-PR55α or CD18/HPAF) had little effect on YAP level and phosphorylation (Fig. 7a, *YAP* and *YAP-S127*: lanes 3–4 and 5–6). In contrast, knockdown of MOB1 in the PR55α-low cells (HPNE-control or CD18/HPAF-PR55α-shRNA) resulted in moderate but noticeable increases in YAP levels (Fig. 7a, *YAP*: lanes 1–2 and 7–8). Furthermore, knockdown of either LATS1 or LATS2 by siRNA only partially compensated for the loss of PR55α, marginally restoring YAP protein level in the PR55α-knockdown cells (see Fig. 7b, *YAP*: lane 6–7 vs. 5). However, such effects are lost when both LATS1 and LATS2 are inhibited by siRNA (see Fig. 7b, *YAP*: lane 8 vs. lanes 5–7), which suggests there might be an alternative YAP inhibitory pathway whose function is activated by the loss of both LATS1/2 and PR55α in the cells. Furthermore, the results of Fig. 7 suggest that the role of PR55α in YAP activation involves both Hippo pathway-dependent and -independent mechanisms, the latter of which could be direct or indirect.

PR55α directed PP2A activity has been shown to positively regulate several oncogenic pathways that play crucial roles in the oncogenesis of solid tumors, namely the Ras/Raf/MEK, Wnt/β-Catenin, and c-Myc signaling pathways^17–19,51,52^. While PR55α activates the Ras/Raf/MEK cascade through dephosphorylating KSR-S392 and RaS259/S295 inhibitory sites that block the pathway, its activation of β-Catenin and c-Myc is via the direct dephosphorylation of β-catenin-T41/S37/S33 and c-Myc-T58, respectively, preventing their proteasomal degradation by β-TrCP. Furthermore, recent studies indicate that cross-talk exists among the PR55α-promoted oncogenic pathways, such as^1^ the Ras/Raf/MEK/ERK signaling that promotes the activation of YAP and c-Myc by increasing their expression^2^, β-catenin that synergizes with YAP/TAZ during cancer progression, and^3^ YAP that is required for KRAS-driving pancreatic tumorigenesis and can compensate for the loss of oncogenic KRAS in the KRAS-addicted pancreatic cells to sustain the malignant phenotypes^25–27,53–55^. These comprehensive data further highlight the significance of PR55α in tumor promotion and the potential of PR55α as a therapeutic target for cancer treatment.

## Materials and methods

### Cell culture and treatment

Human cancer cell lines AsPC-1, Capan-1, CD18/HPAF, L3.6, HeLa, and SH-SY5Y were obtained from ATCC. HPNE is a line of primary human pancreatic ductal cells immortalized by human telomerase hTERT^35^.

Proteasome inhibitor MG132 (EMD Biosciences) was dissolved in DMSO and cells treated at 10 μM^56,57^. Protein synthesis inhibitor CHX (Sigma-Aldrich) was dissolved in water and cells treated at 15 μg/ml^58^.

Cytoplasmic and nuclear extracts were isolated using NE-PER™ Nuclear and Cytoplasmic Extraction Reagents (Thermo Fisher Scientific). Lamin A/C and α-tubulin were used as loading controls for nuclear and cytoplasmic extract, respectively^59^.

Additional details of cell culture/treatment are described in Supplementary Materials.

### Antibodies

Antibodies are listed in Supplementary Materials.

### Immunoblotting and immunoprecipitation

Immunoblotting and immunoprecipitation are described in Supplementary Materials^20,41,60^.

### Short interfering RNA (siRNA) transfection

ON-TARGETplus SMARTpool of siRNA duplexes (Dharmacon) were used for silencing LATS1, LATS2, MOB1A, and MOB1B. Control siGENOME nontargeting siRNA (Dharmacon) was designed to target no known genes in human, mouse or rat. The siRNA sequences are described in Supplementary Materials.

Cells were transfected with 100 nmol/L of siRNA by Dharma*FECT-1* (Thermo Fisher Scientific) as instructed by the manufacturer.

### shRNA lentiviral vectors and viral infection

Dox-inducible lentiviral vector (TRIPZ) expressing shRNAs (Dharmacon) were used. shRNA sequences, lentiviral production, and viral infection are described in Supplementary Materials.

### Retroviral vectors and viral infection

pRevTet-On retroviral vector (Clontech) expresses the reverse tetracycline-controlled transactivator (rtTA) from the CMV promoter. pRevTRE retroviral vector (Clontech) expresses a gene of interest from the Tet-response element (TRE), which contains seven direct repeats of the *tetO* operator sequence upstream of a minimal CMV promoter that can be bound by the tTA or rtTA. The pRevTRE-PR55α retroviral vector contains the PR55α full-length cDNA sub-cloned from pBluescript-SK(-) vector by HindIII/ClaI digestion.

Flag-YAP and Flag-YAP(5SA) expression vectors were made respectively using plasmids p2xFlag-CMV2-YAP (Addgene #19045)^61^ and pCMV-flag-YAP-5SA (Addgene #27371)^62^, both of which encode N-terminally Flag-tagged versions of human YAP (NP_001181973). Coding sequences from both vectors were PCR-amplified using Platinum™-Pfx DNA-Polymerase (Thermo Fisher) using forward (5′-GTACGCGTCGACAGTGAACCGTCAGAATTGATCTA-3′; SalI site underlined) and reverse (5′-CATGGAAGATCTCTATAACCATGTAAGAAAGCTT-3′; BglII site underlined) primers. PCR fragments were then cut with BglII and SalI, gel-purified, and inserted into the BamHI/XhoI sites of pLXSH retroviral vector to produce the final constructs pLXSH-Flag-YAP(WT) and pLXSH-Flag-YAP(5SA). The 5SA mutant carries the following mutations eliminating all LATS1/2-phosphorylation sites in YAP: S61A, S109A, S127A/S128A, S131A, S163A/S164A, and S381A^38,42^.

Retrovirus production and infection are described in Supplementary Materials.

### Immunofluorescence and microscopy

IF and microscopy were performed as described^41^ with additional detail in Supplementary Materials.

Images were taken using a Zeiss-810 confocal laser-scanning microscope. Nuclear/cytoplasmic YAP and PR55α and their co-localization were analyzed by ImageJ^63–65^.

### RT-PCR analysis

Total RNA was isolated using the TRIzol RNA-Isolation Reagent (Invitrogen) and analyzed for human ANKRD1, CTGF, CYR61, GAPDH, MOB1A, MOB1B, and Survivin mRNA levels by RT-PCR using the iScript Advanced cDNA Synthesis Kit and SsoAdvanced Universal SYBR Green Supermix (Bio-Rad). The mRNA expressions were normalized with GAPDH-mRNA levels. PCR-primer sequences are listed in Supplementary Materials.

### Statistical analysis

SigmaPlot was used for statistical analyses. Multiple *t*-tests were used for comparison of experimental groups. *P* values ≤ 0.05 were considered significant.

## Supplementary information

Supplemental figure legends

Supplemental Figure S1

Supplemental Figure S2

SUPPLEMENTARY MATERIALS AND METHODS
