# Supplementary material for: PR55α regulatory subunit of PP2A inhibits the MOB1/LATS cascade and activates YAP in pancreatic cancer cells
Source: Oncogenesis. 2019 Oct 28;8(11):63. doi: 10.1038/s41389-019-0172-9 (PMC6817822; doi:10.1038/s41389-019-0172-9)
Supplement: Supplementary file 1 — Supplemental figure legends [file 41389_2019_172_MOESM1_ESM.docx]

**SUPPLEMENTAL FIGURE LEGENDS**

**Supplemental Fig. S1** **Analysis of intracellular levels and colocalization of PR55α and YAP in human pancreatic normal and cancer cells**. **a-b** CD18/HPAF and HPNE cells, with/without expression of PR55α-shRNA and ectopic PR55α respectively, were stained with anti-PR55a (100C1) and anti-YAP (1A12) antibodies, as described in Fig. 6d-e.  Images were taken with a Zeiss-810 confocal laser-scanning microscope and analyzed for cytoplasmic and nuclear levels of PR55α and YAP using ImageJ and SigmaPlot software with methods described previously (1-4). The bar graphs show the average levels of PR55a and YAP in the cytoplasm and nuclei of a hundred individually-measured cells followed by statistical analysis in the SigmaPlot software. **c** The degrees of colocalization of PR55a with YAP was measured by ImageJ using the Pearson’s overlap coefficients. One hundred cells were individually measured and subjected to statistical analysis using the SigmaPlot software.

**Supplemental Fig. S2** **High expression of MOB1A compared to MOB1B in human pancreatic normal and cancer cells**. **a** Co-amplification of MOB1A and MOB1B transcripts. Total RNA isolated from the indicated cell lines were reverse transcribed (+) or not (-) and subjected to PCR with primers designed to co-amplify MOB1A and MOB1B mRNA sequences to produce a 197 bp fragment. **b** Relative levels of MOB1A and MOB1B expression. To distinguish MOB1A and MOB1B sequences, the common 197 bp fragment was digested with restriction enzyme Alul and resolved by electrophoresis on 2% agarose gels. The position of MOB1A and MOB1B digestion products is indicated. The bar graph shows the relative abundance of MOB1A and MOB1B sequences as quantified by ImageJ and normalized with that of the GAPDH mRNA. **c** Validation of siRNA knockdown. CD18/HPAF and HPNE cells were transfected with siRNAs against MOB1A and/or MOB1B, incubated for 48h and analyzed for total MOB1 protein expression by Western blot analysis.

**REFERENCES**

1. Yan Y, Black CP, Cao PT, Haferbier JL, Kolb RH, Spieker RS, et al. Gamma-irradiation-induced DNA damage checkpoint activation involves feedback regulation between extracellular signal-regulated kinase 1/2 and BRCA1. Cancer Res. 2008;68(13):5113-21.

2. Schindelin J, Arganda-Carreras I, Frise E, Kaynig V, Longair M, Pietzsch T, et al. Fiji: an open-source platform for biological-image analysis. Nat Methods. 2012;9(7):676-82.

3. Thomsen R, Christensen MH. MolDock: a new technique for high-accuracy molecular docking. J Med Chem. 2006;49(11):3315-21.

4. Xie S, Bahl K, Reinecke JB, Hammond GRV, Naslavsky N, Caplan S. The endocytic recycling compartment maintains cargo segregation acquired upon exit from the sorting endosome. Molecular biology of the cell. 2016;27(1):108-26.
