# Supplementary material for: PR55α regulatory subunit of PP2A inhibits the MOB1/LATS cascade and activates YAP in pancreatic cancer cells
Source: Oncogenesis. 2019 Oct 28;8(11):63. doi: 10.1038/s41389-019-0172-9 (PMC6817822; doi:10.1038/s41389-019-0172-9)
Supplement: Supplementary file 4 — SUPPLEMENTARY MATERIALS AND METHODS [file 41389_2019_172_MOESM4_ESM.docx]

**SUPPLEMENTARY MATERIALS AND METHODS**

**Cell culture and treatment**

Human pancreatic cancer cell lines AsPC-1, Capan-1, CD18/HPAF and L3.6 were maintained in Dulbecco’s Modified Eagle’s medium containing 10% fetal bovine serum (FBS). HPNE cells were maintained at 5% CO2 in Medium D growth medium [3 parts high glucose DMEM (GE Healthcare Life Sciences, Pittsburgh, PA) to 1 part M3F (INCELL, San Antonio, TX) supplemented with 10% FBS with 10ng/mL EGF (Invitrogen)] (1). The 293T cell line is a highly transfectable derivative of the human embryonic kidney containing the SV40-T antigen. HeLa cells are human cervical cancer cells. The HeLa cell line was maintained in Dulbecco’s Modified Eagle’s Medium containing 10% FBS. SH-SY5Y is a human neuroblastoma cell line which was maintained in DMEM/F12 media with 10% FBS.

For proteasome inhibition studies, cells were incubated in medium containing 25μM MG132 (EMD Biosciences, San Jose, CA) dissolved in DMSO, as described previously (2, 3), and incubated for the times indicated. For protein half-life assessment, cells were incubated with 15μg/ml cycloheximide (Sigma-Aldrich) for the indicated times and analyzed for protein expression by immunoblotting.

**Antibodies**

All antibodies were obtained from Cell Signaling Technology (Danvers, MA) unless otherwise indicated. These included mouse IgG for Flag (M2) (#3165, Sigma-Aldrich), GAPDH (6C5) (Santa Cruz Biotechnology, Santa Cruz, CA), Lamin A/C (MAB3211) (EMD Millipore, Billerica, MA), LATS2 (kpm) (C-2) (Santa Cruz Biotechnology), MOB1A (G7) (Santa Cruz Biotechnology), PR55 (2G9) (EMD Millipore), PP2A-C subunit (1D6) (EMD Millipore) and YAP (G-6) (Santa Cruz Biotechnology); and rabbit IgG for LATS1 (C66B5), MOB1 (#3863), MST1/STK4 (A300-4651) (Bethyl Laboratories, Montgomery, TX), MST2/STK3 (A300-467A, Bethyl Laboratories), phospho-LATS1(Ser909)/LATS2(SerS872), phospho-LATS1(Thr1079)/LATS2(Thr1041) (D57D3), phospho-MOB1(Thr35) (D2F10), phospho-MST1(Thr183)/MST2(Thr180) (#3681), phospho-YAP(Ser127) (D9W2I), phospho-PR55 (100C1), PP2A-A subunit (PR65) (H-300) (Santa Cruz Biotechnology) and YAP(Ser397) (D1E7Y). Secondary antibodies for immunofluorescence studies included Alexa Fluor 594 Donkey Anti-rabbit IgG (H+L) and Alexa Fluor 488 Donkey Anti-mouse IgG (H+L) from Thermo Fisher Scientific. Nuclei were visualized by DAPI (Sigma-Aldrich, St. Louis, MO) staining at 1µg/ml for 5 min.

**Immunoblotting and immunoprecipitation**

Immunoblotting (IB) and immunoprecipitation (IP) were performed as described previously (4-6). Specific protein signals on Western blots were visualized by chemiluminescence exposed to x-ray film, scanned using EPSON Perfection 4490PHOTO scanner and analyzed with ImageJ (NIH, Bethesda, MD) and SigmaPlot (version 11.2) software. For the co-IP study, PR55α and YAP were immunoprecipitated from cell lysate using anti-PR55α and anti-YAP antibody, respectively, and the precipitates probed for the presence of both PR55α and YAP, as well as PP2A-A and PP2A-C subunits by Western blot analysis with specific antibodies.

**Short interfering RNA transfection**

The sequence of the control siRNA is 5’-UAAGGCUAUGAAGAGAUAC-3’. SMARTpool siRNA for LATS1, LAT2, MOB1A, and MOB1B each contains four siRNAs, targeting multiple sites on the mRNA of interest. The sequences for LATS1 siRNA are 5’-GGUGAAGUCUGUCUAGCAA-3’, 5’-UAGCAUGGAUUUCAGUAAU-3’, 5’-GGUAGUUCGUCUAUAUUAU-3’ and 5’-GAAUGGUACUGGACAAACU-3’. The sequences for LATS2 siRNA are 5’-GCACGCAUUUUACGAAUUC-3’, 5’-ACACUCACCUCGCCCAAUA-3, 5’-AAUCAGAUAUUCCUUGUUG-3’ and 5’-GAAGUGAACCGGCAAAUGC-3. The sequences for MOB1A are 5’-GAAGCAAGCUGUCCAGUCA-3’, 5’-GCAGAUGGUACUAAUAUUA-3’, 5’-CAAAGACUAUUCUAAAGCG-3’ and 5’-AAUCUGAGACAAGCUGUUA-3’. The sequences for MOB1B siRNA are 5’-GCAGAUGGAACGAACAUAA-3’, 5’-GAGGAAGCACAUCUAAAUA-3’, 5’-GCUCUGCACCAAAGUAUAU-3’ and 5’-AUGAAUGGGUUGCAGUUAA-3’.

Cells were transfected with 100 nmol/L of siRNA and incubated for 48-72h. All cell lines were transfected with Dharma*FECT-1* (Thermo Fisher Scientific, Waltham, MA).

**shRNA lentiviral vectors and viral infection**

shRNAs targeting various regions of the PR55α (*PPP2R2A*) gene are 5'-ACTGTAGAAATTATATCTG-3', 5’-ATGGCTAGCAGACATGGAG-3’, 5’- ATTTCATCTTTCTTTCGCT-3, 5’- TGGATGAAATTCTGCTGCT-3’ and 5’- CAACTATCTCAACTAAGCA-3’. The sequence for non-silencing Control-shRNA is 5’‑ ATCTCGCTTGGGCGAGAGTAAG-3’.

shRNA lentiviral expressing vectors were packaged in HEK 293T cells using the ViralPack Transfection Kit (Agilent Technologies, Santa Clara, CA), according to the manufacturer’s instruction. At 24, 48 and 72 h post-transfection, medium containing lentivirus was collected, filtered through a 0.4 μM filter and cells infected in the presence of 4 μg/ml polybrene (Sigma-Aldrich). Cell populations stably expressing shRNAs were selected in medium containing 1-2 μg/ml puromycin (Sigma-Aldrich). The levels of PR55α in the selected clones were determined by Western blot analysis.

**Retroviral vectors and viral infection**

Retrovirus [pRevTet-On, pRevTRE, pRevTRE-PR55α, pLXSH-Flag, pLXSH-Flag-YAP(WT) and pLXSH-Flag-YAP(5SA)] was produced using Phoenix A retroviral packaging cells as described in our publication (5). At 48h post-transfection, the retrovirus-containing medium was collected, filtered through a 0.4 µm filter and used for cell infection. To construct HPNE cells expressing rtTA, cells were first infected with the pRevTet-On retrovirus (10 pfu/cell) in the presence of 4 µg/mL polybrene and then selected for 2 weeks in medium containing 600 µg/ml G418. Doxycycline-regulated clones (HPNE-TetOn) were then identified by a luciferase reporter assay using the pTRE-Luc vector (Clontech), as instructed by the manufacturer. Subsequently, the HPNE-TetOn cells were infected with pRevTRE-PR55α (or pRevTRE-control) retrovirus and selected with 200 µg/mL hygromycin (Clontech). The obtained clones were validated by Western blot analysis for the induction of PR55α by Dox. To construct CD18/HPAF cells expressing Flag-YAP(WT) and Flag-YAP(5SA), the cells were infected with retroviral vector pLXSH-Flag, pLXSH-Flag-YAP(WT) and pLXSH-Flag-YAP(5SA), selected with 200 µg/mL hygromycin and positive clones were validated by Western blot analysis.

**Immunofluorescence and microscopy**

Cells were grown on coverslips (thickness #1) and fixed in 4% paraformaldehyde for 20 minutes, followed by incubation with 0.25% Triton X-100 for 10 min to achieve permeabilization. Samples were then blocked for 30 min using 10% horse serum, 1% bovine serum albumin and 0.5% Tween-20 in TBS at ambient temperature. Cells were then incubated overnight at 4**°**C in mouse anti-YAP antibody (1:50 dilution, Cat. # 376830, Santa Cruz) and rabbit anti-PR55 antibody (1:100 dilution, Cat. #4953, Cell Signaling) in TBS with 0.05% Tween-20. After washing with 1% BSA in TBS, cells were incubated with Alexa Fluor 594 donkey anti-rabbit IgG (1:200 dilution, Cat. #A-21207) and Alexa Fluor 488 donkey anti-mouse IgG (H+L), (1:200 dilution, Cat. # A-21202) for 1 hour. DAPI staining occurred at a 1:1000 dilution for 5 min. Images of immunostained cells were taken using a Zeiss 810 confocal laser-scanning microscope from the UNMC Microscopy Core Facility.

**Quantitative RT-PCR analysis**

The PCR primer sequences are: ANKRD1, 5'-GTGTAGCACCAGATCCATCG-3' and 5'-CGGTGAGACTGAACCGCTAT-3'; CTGF, 5'-TTGGCAGGCTGATTTCTAGG-3' and 5'-GGTGCAAACATGTAACTTTTGG-3'; CYR61, 5'-CCCGTTTTGGTAGATTCTGG-3' and 5'-GCTGGAATGCAACTTCGG-3'; GAPDH, 5’-AACAGCGACACCCACTCCTCC-3’ and 5’-CATACCAGGAAATGAGCTTGACAA-3’; MOB1A, 5’- GTGAACACTGTGGATTTCTT-3’ and 5’-TCTTGAACCCAAGTCATCAA-3’; MOB1B, 5’-GTTAACACTGTGGATTTCTT-3’ and 5’-TCCTGAACCCAAGTCATCAA-3’; and Survivin, 5'-CGAGGCTGGCTTCATCCACT-3' and 5'-ACGGCGCACTTTCTTCGCA-3'.

**REFERENCES**

1. Lee KM, Nguyen C, Ulrich AB, Pour PM, Ouellette MM. Immortalization with telomerase of the Nestin-positive cells of the human pancreas. Biochem Biophys Res Commun. 2003;301(4):1038-44.

2. Choudhury AD, Xu H, Baer R. Ubiquitination and proteasomal degradation of the BRCA1 tumor suppressor is regulated during cell cycle progression. J Biol Chem. 2004;279(32):33909-18.

3. Chen F, Zhang Z, Bower J, Lu Y, Leonard SS, Ding M, et al. Arsenite-induced Cdc25C degradation is through the KEN-box and ubiquitin-proteasome pathway. Proc Natl Acad Sci U S A. 2002;99(4):1990-5.

4. Hein AL, Seshacharyulu P, Rachagani S, Sheinin YM, Ouellette MM, Ponnusamy MP, et al. PR55alpha Subunit of Protein Phosphatase 2A Supports the Tumorigenic and Metastatic Potential of Pancreatic Cancer Cells by Sustaining Hyperactive Oncogenic Signaling. Cancer Res. 2016;76(8):2243-53.

5. Yan Y, Black CP, Cao PT, Haferbier JL, Kolb RH, Spieker RS, et al. Gamma-irradiation-induced DNA damage checkpoint activation involves feedback regulation between extracellular signal-regulated kinase 1/2 and BRCA1. Cancer Res. 2008;68(13):5113-21.

6. Yan Y, Cao PT, Greer PM, Nagengast ES, Kolb RH, Mumby MC, et al. Protein phosphatase 2A has an essential role in the activation of gamma-irradiation-induced G2/M checkpoint response. Oncogene. 2010;29(30):4317-29.
